# Cemented vs. Cementless Fixation in Primary Knee Replacement: A Narrative Review

**DOI:** 10.3390/ma17051136

**Published:** 2024-02-29

**Authors:** Mikołaj Wilczyński, Michał Bieniek, Przemysław Krakowski, Robert Karpiński

**Affiliations:** 1 Orthopaedic and Sports Traumatology Department, Carolina Medical Center, Pory 78, 02-757 Warsaw, Poland; mikolaj.wilczynski@carolina.pl (M.W.); michalbieniek7@gmail.com (M.B.); 2 Department of Trauma Surgery and Emergency Medicine, Medical University of Lublin, Staszica 11, 20-081 Lublin, Poland; 3 Department of Machine Design and Mechatronics, Faculty of Mechanical Engineering, Lublin University of Technology, Nadbystrzycka 36, 20-618 Lublin, Poland

**Keywords:** bone cements, knee, total knee replacement (TKR), total knee arthroplasty (TKA), osteoarthritis

## Abstract

Knee osteoarthritis (OA) is one of the leading causes of disability around the globe. Osteoarthritis is mainly considered a disease affecting the elderly. However, more and more studies show that sports overuse, obesity, or congenital disorders can initiate a pathologic cascade that leads to OA changes in the younger population. Nevertheless, OA mostly affects the elderly, and with increasing life expectancy, the disease will develop in more and more individuals. To date, the golden standard in the treatment of the end-stage of the disease is total joint replacement (TJR), which restores painless knee motion and function. One of the weakest elements in TJR is its bonding with the bone, which can be achieved by bonding material, such as poly methyl-methacrylate (PMMA), or by cementless fixation supported by bone ingrowth onto the endoprosthesis surface. Each technique has its advantages; however, the most important factor is the revision rate and survivor time. In the past, numerous articles were published regarding TJR revision rate, but no consensus has been established yet. In this review, we focused on a comparison of cemented and cementless total knee replacement surgeries. We introduced PICO rules, including population, intervention, comparison and outcomes of TJR in a PubMed search. We identified 783 articles published between 2010 and 2023, out of which we included 14 in our review. Our review reveals that there is no universally prescribed approach to fixate knee prostheses. The determination of the most suitable method necessitates an individualized decision-making process involving the active participation and informed consent of each patient.

## 1. Introduction

A total knee replacement is the treatment of choice in end-stage osteoarthritis (OA). There is no single explanation of OA development, and multiple factors, such as obesity, genetics, trauma or excessive sport, have been studied in the past as causes of the disease [1,2,3,4,5]. At first, osteoarthritis was presented as a simple degenerative process of “wear and tear”. Nowadays, the most popular theory is based on the inflammatory factor. The inflammatory process starts in the synovial membrane with the activation of the autoimmune system. The inflammatory cytokines and cells slowly destroy cartilage and then the subchondral bone. In the final stage, soft tissues like the joint capsule, menisci and ligaments break down [6,7,8]. Cytokines such as matrix metalloproteases (MMP) are involved in extracellular matrix degradation with the predominant role of MMP-13 and MMP-1, which both induce collagen break down [9]. Other cytokines, such as IL-1 or IL-6, are also involved in the inflammatory response of the joint, which further impedes joint function and promotes derangement of vital joint tissues such as the synovium, cartilage, ligaments or menisci [10].

The clinical presentation of OA differs among patients; however, the main symptoms include the following: pain, swelling, reduced range of motion or stiffness, subchondral bone remodeling. In the beginning, the pain appears during the activity and slowly disappears after a few minutes. However, if not treated properly, the OA changes progress, and symptoms that firstly were mild and transient become prevalent and severe and do not resolve after rest [11,12].

The diagnosis is mainly based on symptoms and imaging modalities. Typical radiological characteristics include narrowing of the joint space, sclerosis and cyst formation in the subchondral bone layer and osteophytes on conventional radiography [13,14]. Other imaging modalities, such as magnetic resonance imaging, computed tomography or alternative methods, such as vibroarthrography [15,16,17] of the joint, can be utilized in the diagnostic route. However, conventional radiography still remains the gold standard.

To date, there is no successful approach for restoring articular cartilage (AC) and, therefore, treating osteoarthritis. Multiple surgical interventions have been proposed in the literature; however, to date, there is no study showing hyaline cartilage regeneration in vivo. This is one of the biggest challenges of modern orthopedics [18,19,20].

Among the risk factors, such as obesity, age, muscle imbalance, factors related to labor, sport activity, congenital structural disorders, genetic factors or female sex [21,22,23,24], which accelerate the development of OA, age is one of the most important non-modifiable factors. Almost 30% of the population over 45 years of age has some features of osteoarthritis [25]. Over 80% of people over 55 present radiological symptoms of OA; however, they can remain symptomless for many years [25,26,27,28]. Nevertheless, it is very important to diagnose osteoarthritic changes in the early stage in order to start treatment early enough to postpone the necessity of knee replacement surgery [29].

In the early stages of the disease, conservative treatment is introduced to relieve the symptoms and prolong knee function. These modalities include functional physiotherapy and various medications like NSAIDs, steroids, hyaluronic acid injections, platelet-rich plasma injections, glucosamine or chondroitin supplementation [30,31,32,33,34]. If conservative measures fail, surgical treatment is introduced. In cases of unilateral disease, there is an option for an osteotomy or unicompartmental knee replacement [35,36,37,38]. Patients diagnosed with osteoarthritis of both compartments must undergo a total knee replacement.

While imaging’s diagnostic role in osteoarthritis is restricted, studies demonstrate its significance as a predictor for the success of joint replacement, suggesting a potential crucial role in surgical referral decisions [39]. For advanced osteoarthritis, joint replacement surgery proves both clinically effective and cost-efficient [40,41]. If conservative treatment fails to alleviate symptoms in patients with advanced radiographic findings over 3 months and there is a significant disease burden, joint replacement may be considered. Before surgery, the treating surgeon must assess the contraindications, discuss patient expectations and optimize modifiable risk factors affecting the outcome [42]. The most common causes of patient dissatisfaction after a total knee arthroplasty (TKA) are persistent pain and functional limitations, but despite this, Halawi et al. reported that 88% of patients feel satisfied because of the procedure. Moreover, pain was the reason for dissatisfaction in 41% of patients, functional limitation in 26% and surgical complications/reoperations in 12% [43]. Advanced age is not a contraindication to TKA because the procedure has been shown to significantly improve function and quality of life without an overall impact on morbidity and mortality [44].

Osteoarthritis is mainly considered a disease affecting the elderly; however, more and more studies show that sports overuse, obesity or congenital disorders can initiate a pathologic cascade that may lead to osteoarthritis changes in the younger population [45,46]. There is evidence that the risk of developing osteoarthritis is influenced not only by current body weight, but also by maximum lifetime BMI, and that this influence is greater than current body weight [47]. Nevertheless, osteoarthritis mostly affects the elderly, and with an increasing life expectancy, the disease is expected to develop in more and more individuals.

Due to a rising young population in need of TKA and a greater number of performed surgeries, standard cemented knee protheses were linked to a range of complications typical for this kind of patient. Increasing rates of aseptic tibial loosening, revisions and replacements urged the manufacturers to invent a cementless fixation and introduce the idea to orthopedic practitioners [48]. The cementless knee replacement has both advantages and disadvantages as compared to the classic system. Although the new idea was met with great enthusiasm at the beginning, more and more defects have been shown in the literature [49], and the majority of surgeons came back to the standard cemented fixation of total knee arthroplasty.

Currently, the most important factors for choosing the appropriate type of fixation are the revision rate and survivor time. In the past, numerous papers were published regarding the TKR revision rate, but no consensus has been established yet [50,51]. Some papers suggest that cementless fixation can produce a better survivor rate in younger or obese patients, whereas a cemented TKA is believed to have less infectious complications. However, in the literature, research showing no statistical difference in survivor rate at a 10-year follow-up between cemented and cementless TKA, regardless of age or BMI can be found [52]. To date, there is no strong consensus regarding which patients should receive a cemented or cementless TKA. However, the type of fixation influence on the risk of revision and the survival time of the prosthesis must also be discussed. In this review, the authors focus on a comparison of cemented and cementless total knee replacement.

## 2. Materials and Methods

**Figure 1:**
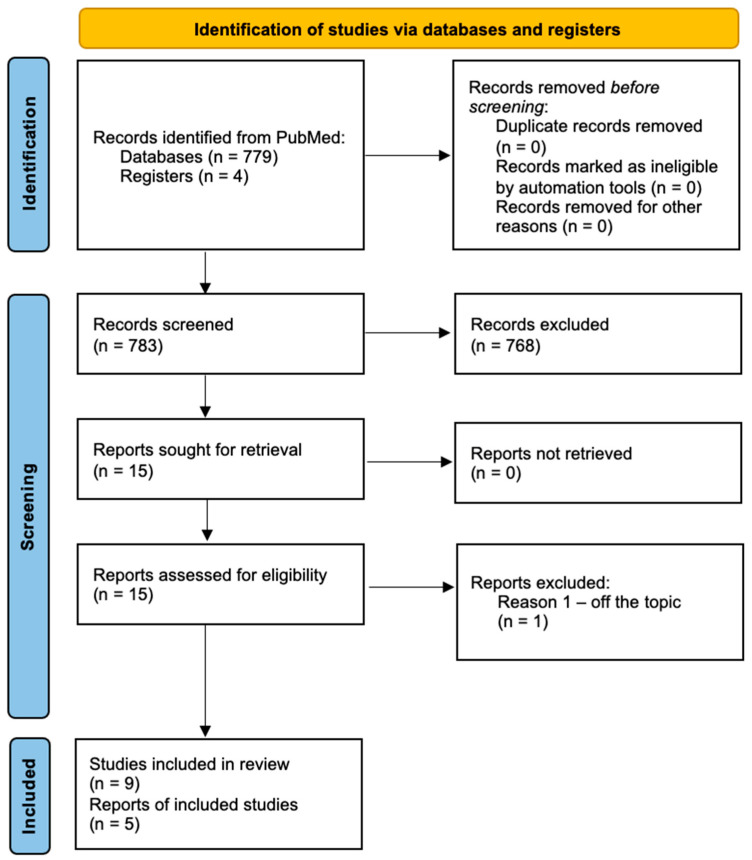
Flow diagram of included studies—PRISMA 2020.

The review was performed by searching the PubMed electronic database for articles published between 2010 and 2023. The search was performed simultaneously by two authors (MW, MB). The authors introduced PICO rules, including population (primary knee replacement, primary knee arthroplasty, primary TKR/TKA/UKA/UKR), intervention (cemented knee arthroplasty, cemented knee replacement, cemented TKR/TKA/UKA/UKR), comparison (cementless knee arthroplasty, cementless knee replacement, uncemented knee replacement, uncemented knee arthroplasty, uncemented/cementless TKR/TKA/UKA/UKR) and outcomes of a TKR (knee revisions, knee revision time, knee reoperation, knee reoperation time) in a PubMed search and identified 779 records. The inclusion protocol is shown in Figure 1. The search was limited to articles in English and about adult patients. The authors excluded articles describing revision knee replacements and a hybrid type of fixation. Nine studies and one report were included in this review. All the articles were initially screened for their titles and abstracts. The authors additionally searched the reference lists in the included articles. Furthermore, the authors based their assessment on the statistics from four latest National Joint Registries: UK (2023), USA (2023), Australia (2023) and Sweden (2022) (Figure 1 [53]).

## 3. Cemented Knee Replacement

**Figure 2:**
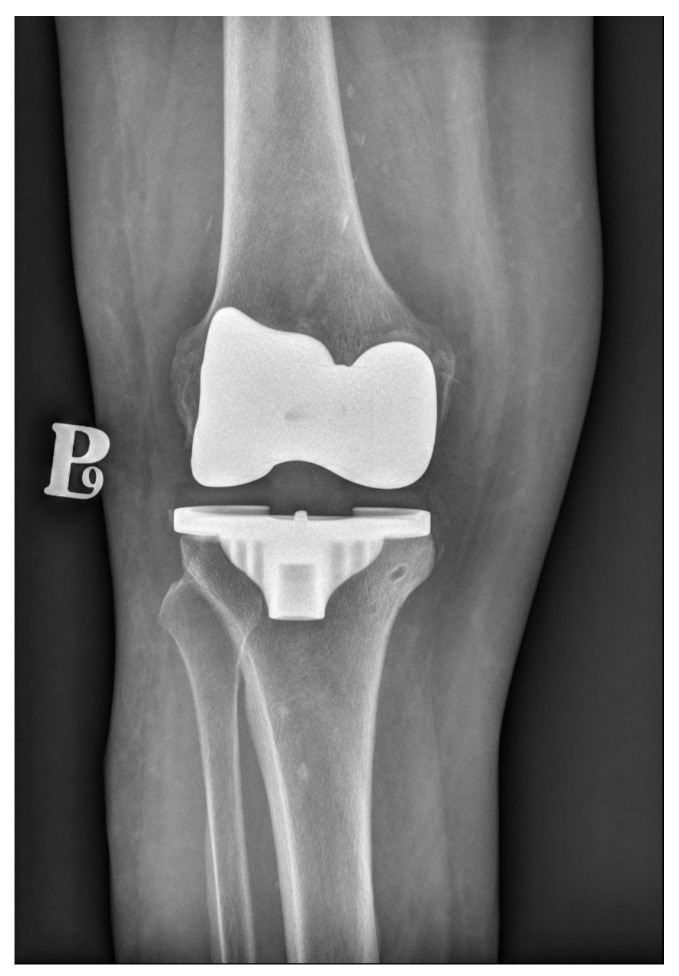
Conventional radiograph presenting cemented total knee replacement.

Knee arthroplasty is one of the most commonly performed orthopedic procedures. There are two main types of fixation methods—cemented (which is shown in Figure 2) and cementless. A third type available is hybrid fixation, which is not very common. In the hybrid fixation, the tibial side is cemented, and the femoral side is cementless. This type of arthroplasty has gained some support from national registries. For example, in the Australian registry from 2022, hybrid TKRs accounted for about 18% of all TKRs, and their 10-year survivorship was superior to full-cemented or cementless TKRs [54]. The cemented type has been the gold-standard since the beginning of performing arthroplasties until today. In reference to the registries included in our review, 77.6% of all primary TKAs in the USA in 2022 were cemented [55].

**Figure 3:**
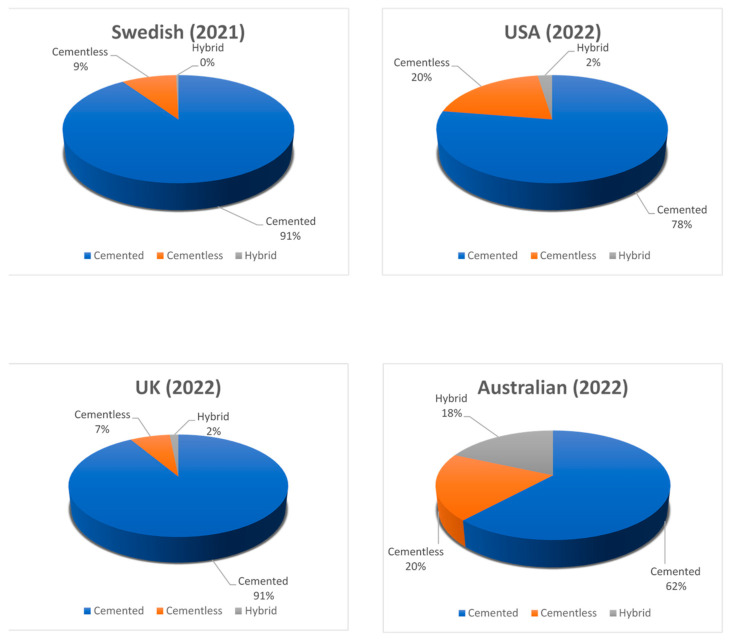
Presentation of different types of fixation used in primary TKR based on national registries.

The Swedish registry states 90.5% of cemented fixation primary TKA in 2021 [56]. The Australian registry reports 61.8% primary cemented TKA in 2022 [57]. In the same year, in the UK, there was 83.1% primary cemented TKA [58]. These statistics show that the cemented type of fixation is still the significant majority despite the increasing popularity of cementless and hybrid types of fixation. Figure 3 presents the percentage breakdown of fixation methods found in the Swedish, Australian, British and American endoprosthesis registries.

Bone cement, or polymethylmethacrylate (PMMA), has been used in surgical fixation of protheses over the last 60 years [59,60,61,62]. The primary function of bone cement is to transfer forces from the bone to the prosthesis [63,64]. The dough formed by mixing ground PMMA powder and a liquid monomer, which hardens when benzoyl peroxide is added and the mixture is heated to 100 °C in a stone mold. The era of modern PMMA bone cements stems from the patent by Degussa and Kulzer (1943), describing how PMMA polymerizes at room temperature if a co-initiator, such as a tertiary aromatic amine, is added [65]. Dental surgeons were the first to use this technology for dental fixations. The first bone cement used in orthopedics is credited to English surgeon, Dr. John Charnley, who used “dental acrylic” in 1958 for a total hip arthroplasty [66].

However, due to the mentioned chemical reactions and the fact that the cement polymerization process is an exothermic reaction, the consequences of using this fixation solution in joint arthroplasty must be taken into account. The high peak curing temperatures of acrylic bone cements can cause tissue necrosis [67,68,69]. Furthermore, Vale et al. noticed that acrylic bone cement induces the production of free radicals by human fibroblasts. This radicals are described in the literature as inflammatory mediators [70,71]. That means that using cement in humans may be cytotoxic [72].

The explanation of the fact that cemented fixation is still used in the majority of surgeries can be found in the literature, in which, over the last decade, multiple advantages of such a fixation method have been described. A cemented TKA allows for greater primary stability. It is a less demanding procedure considering the technique. Moreover, using the cement allows the surgeon to deliver antibiotics locally at the surgery site [48]. Cemented fixation is three times cheaper than uncemented components, which is especially important regarding the national healthcare settings and their limited budget [73]. However, Lawrie et al. reported that the overall procedural cost of implanting a cementless TKA is lower than implanting a cemented TKA [74].

Chiou et al. showed in their retrospective review of over 320,000 patients that compared to cemented TKA, cementless TKA is associated with increased rates of irrigation and debridement procedures, one-component revision and arthroscopy with lysis of adhesions [75]. Moreover, Mohammed et al., in their analysis based on 44,954 patients from the national registry, revealed that in the cementless cohort, the group rates of revision for pain were higher when compared to the cemented group. In addition to this, the cemented group had a better 10-year free of reoperation rate than the cementless group (82.7% vs. 81.4%), which was statistically significant (*p* = 0.001). In addition to this, the risk of medical complications, such as the rates of pulmonary and urinary tract infections, were lower for the cemented group [76]. The study of Edgar et al., based on 168 patients, shows that patients who underwent a cemented TKA required less manipulations under anesthesia and had a higher postoperative range of motion compared to patients undergoing cementless TKA [77]. In the meta-analysis of Mercurio et al., which consisted of 5222 patients, they found cemented TKA showed less blood loss compared to the cementless type of fixation [78]. On the other hand, in the research of Owens et al., they noticed that a history of chronic obstructive pulmonary disease, black race, prolonged operative time, steroid use, bleeding disorder and ASA class 3–4 were independent predictors of the need for blood transfusion, regardless of the type of fixation [79].

What is worth noting in the Swedish 2021 registry is that in TKRs implanted in the period of 1985–1994, when the use of uncemented implants was slightly more common, these had a higher risk of revision. Additionally, in the last ten-year period (2012–2021), despite the evolution of cementless components, there was a significantly higher risk of revision in uncemented implants compared with cemented [56].

**Figure 4:**
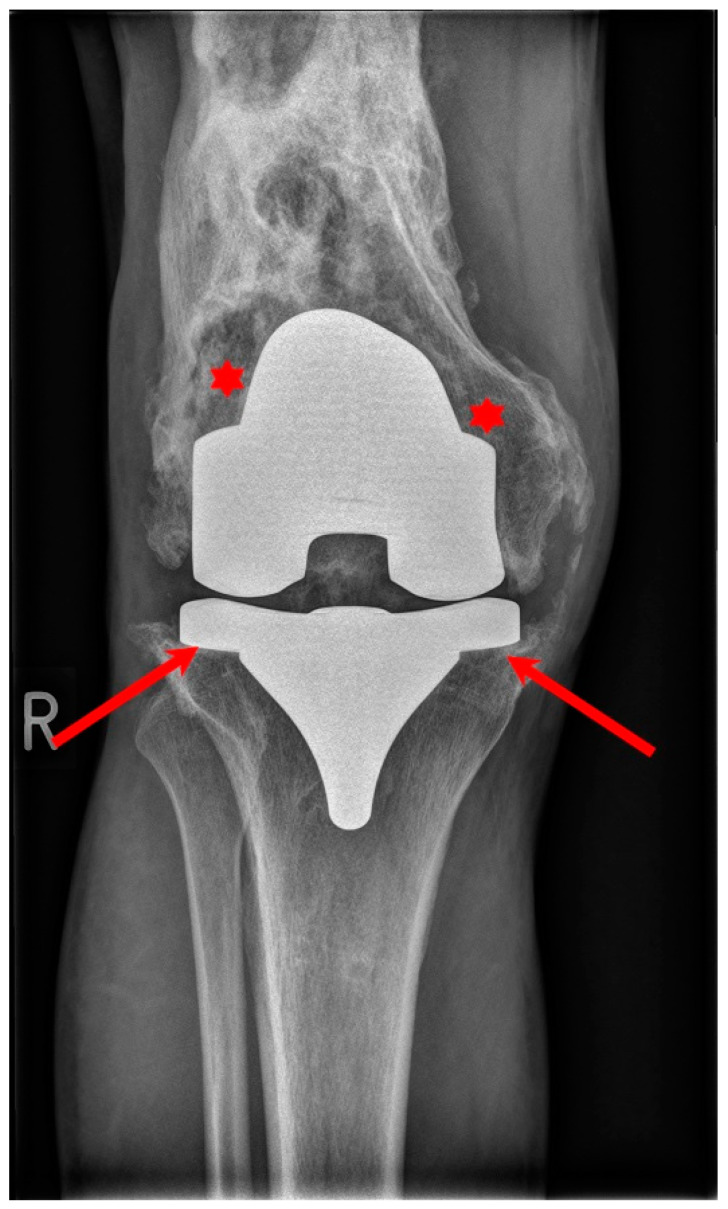
Conventional X-ray showing septic loosening of the TKR with bone destruction (asterisk) and loosening of the implants (arrows).

The use of cement in a TKA requires appropriate prepping, careful application of the cement and removal of excess cement with thorough washing prior to closing. This process adds extra time to the surgery duration and removes natural growth factors, which are important for fast adaptation and fixation of the implanted prothesis. Since a cementless TKA relies on the biological bond between the bone and the implant, this process may be skipped, thus, the length of the procedure is shorter. As longer operative times are associated with a greater chance of postoperative infection, there is a possibility to add an antibiotic into the cement and prevent periprosthetic infection, which is a debilitating complication that can be observed in Figure 4. Furthermore, cemented fixation is technically problematic when revision is needed, making reoperation impossible in some particular cases [48]. During this procedure, cement used in the primary total knee replacement has to be removed, as well as the protheses components themselves. Removing this cement from the bone is a time-consuming process that adds extra time to the length of the surgery. After this, the bone surfaces must be prepared for the revision implant. In some cases, there may be significant bone loss around the knee. If this occurs, metal augments and platform blocks could be necessary to add to the main components in order to provide suitable fixation. Sometimes bone graft materials can be used as well.

It is important for the surgeon and the patient to carefully consider both methods of fixation before the surgery, taking into account the advantages and disadvantages of cemented fixation knee replacement.

## 4. Cementless Knee Replacement

The first considerations about cementless TKA appeared in the mid-1980s [80]. Early cementless TKA designs resulted in failure due to increased wear and osteolysis, but the design has now improved [48]. First, cementless prostheses were made of stainless steel and, later, of cobalt chrome, which increased stiffness and wear resistance [81]. The integration of a hydroxyapatite (Ca_10_[PO_4_]_6_[OH]_2_) coating marked a significant advancement in the overall composition of the implant [82]. Søballe et al. reported that implants coated with hydroxyapatite have greater interface shear strength compared with titanium without hydroxyapatite [83]. The roughness surface of cementless implants is important because that facilitates osseointegration and provides mechanical interlock prior osseointegration [84]. Some recent publications have shown that the use of improved biomaterials, including porous metals such as porous tantalum and highly porous titanium, reported better outcomes [85]. In addition to osseointegration and enhanced interface strength, porous tantalum offers crucial biomechanical advantages stemming from its increased material elasticity and a high surface coefficient of friction. Porous tantalum’s optimal elasticity offers a potential solution to the issue of stress shielding in orthopedic implants by enabling a more physiologic transfer of stresses to the periprosthetic bone [86]. Nowadays, the majority of cementless TKA implants are made with a porous plasma-sprayed (PPS) tibia or 3D printing [82]. Another option to improve the results of a cementless prosthesis with theoretical more precision in the bone preparation that will allow for improved fixation may be a robotic-assisted TKA [87]. Each year, the number of cementless TKAs is increasing. Based on the American registry, 20% of all primary TKAs in 2022 were cementless [55]. Similar information was reported in the Swedish register. Between 2010 and 2021, the number of cementless TKAs increased from 2.4% to 9.1% [56]. The Australian registry reports that in 2018, the rate of cementless primary TKAs was 8.3%, and in 2022, this rate increased to 19.9% [57]. In the UK, opposite statistics were reported because the rate of cementless and hybrid TKAs decreased between 2004 and 2022 from 9% to 2.3% [58].

Cemented fixation remains a gold standard [88] in TKA. However, especially in younger, obese and active patients, it has shown an increased failure rate due to the greater stress placed on the bone–implant interface [50], which brings research interest into cementless arthroplasty. It is important to note that cementless arthroplasty procedures are shorter than those that use cement fixation [85], hence patients who have a cementless TKA have a lower tourniquet time than patients who have a cemented TKA [77]. A study by Olivecrona et al. shows that a longer tourniquet time was associated with a higher risk of complications after knee arthroplasty, and special attention is advocated to reduce the tourniquet time [89].

An arthroplasty without cement allows for greater preservation of bone and an easier revision procedure, if required [90]. Cementless arthroplasty eliminates complications related to cemented TKA, like thermal necrosis caused by cement polymerization [91,92].

Historically, knee arthroplasty was mostly performed on elderly patients. A Swedish report shows that the number of patients under 65 undergoing arthroplasty has now increased [56]. Based on American reports, cementless fixation for primary TKA is associated with a reduced rate of cumulative percent revision in men of all ages (<65, 65 and older) but a significantly increased rate in women aged 65 and older. There was no significant difference in young females (<65) [55]. Wang et al. showed in their meta-analysis based on 510 TKAs that compared to cement fixation, cementless TKA gives better clinical outcomes in patients aged 65 years or younger; however, the survival rate and complications were similar between these groups. Primary cementless TKA in a young group reported a higher KSS functional score, better ROM recovery and fewer radiolucent lines (<1 mm) [93]. The gold standard in diagnostic aseptic loosing is conventional radiography. Radiolucent lines at the cement–bone or metal–cement interface of more than 2 mm are a sign of loosening [94]. Mohammed et al. also revealed that cementless components can have some advantage in a young group (<55), such as a lower revision rate for aseptic loosening. Moreover, in a cementless cohort group, the rates of revision for infection were lower when compared to the cemented group, and the rates of wound dehiscence were lower in cementless arthroplasty [76]. Furthermore, in the meta-analysis of Mercurio et al., they found that cementless arthroplasty has a lower rate of aseptic loosening [78]. Moreover, based on the British registry, revision incidences for TKA that were uncemented were lower than cemented TKA for infection. The same was true for periprosthetic fracture but the rates were higher for all the other recorded indications [58]. Table 1 summarizes the papers included in this paper.

## 5. Discussion

The preferred technique for fixation TKA remains a topic of discussion. While cemented fixation is considered the gold standard with outstanding long-term outcomes, it falls short of ideal longevity in younger, obese and more active individuals. Cementless TKA presents a potential solution by providing the chance to achieve biological fixation and addressing these limitations [95].

The first cementless TKAs, which were performed in mid-1980s, had many disadvantages. Nowadays, the technique and prosthesis have improved, and demographic changes have led to more extensive use of cementless fixation [48]. There are several articles that compare cemented TKA and cementless TKA. Prasad et al. reported in their meta-analysis including 755 TKAs that there is no significant difference in the revision rate or postoperative functional knee scores between cemented and cementless TKA implants up to 16.6 years follow-up [84]. Moreover, Kim et al. revealed in their trial based on 261 patients (522 TKAs) that cementless TKA and cemented TKA have comparable outcomes and survivorship in young patients [96]. In the meta-analysis of Mercurio et al., they found that when cemented and cementless fixations are compared in primary TKA, comparable functional outcomes can be achieved [78]. Wang et al. proved that complication and survival rates were similar between different fixation types groups, as well [93]. In the research of Mohammad et al., the 10-year implant survival was 95% in both the cemented and cementless cohort groups [76]. However, in a Swedish report, cementless TKA was linked to a higher risk of revision rate [56]. The difference in revision rates may be due to the experience of the surgeon. Achieving success with a cementless TKA requires a careful surgical approach, allowing a minimal margin for error and requiring precise bone resections. A cemented TKA enables the filling of minor resection defects with the cement mantle [48]. Mohammed et al. reported in their analysis based on 44,954 patients from the national registry that the occurrence of periprosthetic fractures after cemented and cementless TKA surgeries is minimal, with rates around 1.2% and 1.4%, respectively, over a 10-year period. There were no significant differences in the rate of periprosthetic fractures requiring rehospitalization between patients who underwent cemented and uncemented TKA [97]. The same data were reported in the updated UK report [58]. Two of the included articles and the National Joint Registry from the UK presented that cementless TKA has a lower risk of revision due to infection [58,76,78]. However, the American registry, based on the data collected between 2012 and 2022, shows no significant differences between fixation cohorts for primary total knee arthroplasty when evaluating revision for infection in patients 65 years of age and older [55]. Two of the included papers examined the rate of postoperative pain and revision for this reason. Prasad et al. noticed an increased postoperative score pain in the cementless fixation group [84]. Moreover, Mohammed et al. presented a conclusion that in the cemented group, the revision rates due to pain were lower [76]. This could be explained by the fact that early loosening was not seen on X-rays. However, revisions due to pain are poorly defined and can be caused by many reasons not related to loosening alone [98]. Interestingly, among these numerous conclusions, which are rather aligned, the results of two particular studies are contradictory. Edgar et al., in their article on 168 patients, revealed lower rates of manipulations under anesthesia and a greater final range of motion in patients who underwent a cemented TKA [77]. Against this, Mercurio et al. presented in their meta-analysis based on 5222 patients a lower rate of manipulation under anesthesia in patients who underwent a cementless TKA [78]. On the other hand, the research of Rahman et al., consisting of 524 patients, was focused on the results of cemented and cementless fixation of unicompartmental knee replacement (UKR). They determined that UKR has remarkably lower pain levels compared to the TKR scores reported in the literature. They also concluded significantly less pain in cementless UKR compared to cemented UKR [99]. Based on our findings, cementless fixation should be considered in younger, more active or obese patients, since it shows better survivor time in this group of patients, whereas cemented fixation should be considered in older patients with a higher risk of infection.

It should be taken into consideration that it is not only the type of fixation that affects the risk of revision and the survival time of the prosthesis. Law et al. reported that the risk of revision after TKA is higher and implant survival is decreased if the patient uses cannabis [100]. However, Denduluri et al. reported that in a 6-year follow-up group, cannabinoid use did not appear to be associated with increased perioperative complications, considering that during this period, cannabinoid use increased more than 60% and opioid use decreased approximately 30% [101]. On the other hand, chronic use of opioid medications prior to a total knee arthroplasty may lead to a substantially greater risk for complications and painful prolonged recoveries. The authors believe this may be related to drug dependence or hyperalgesia, both of which might influence perioperative and postoperative pain management, rehabilitation and clinical outcomes after a total knee arthroplasty. Alternative non-opioid pain medications or earlier referral to an orthopedic surgeon should be considered for patients with painful knee osteoarthritis [102]. In addition, Hernandez et al. stated that excessive use of opioids prescribed after a total knee replacement should be avoided because there was no difference in pain relief between small and large doses of opioids [103]. 

Chronic systemic diseases also affect the revision rate and implant survival time after TKA. Watts et al., in their review, stated that insulin dependence further increased the risk of reoperation, revision and deep infection in morbidly obese patients [104]. Furthermore, Sloan et al. reported that hypoalbuminemia appears to be a more significant risk factor for readmission and reoperation than even the highest categories of obesity [105]. Additionally, patients infected with hepatitis C virus have an increased risk of surgical complications and higher rates of readmissions and reoperations, especially due to periprosthetic infections [106,107,108,109]. Interestingly, Tay et al. noticed that comorbidities have a greater impact on TKA outcomes than age in an octogenarian cohort group of patients [110]. Ottesen et al. proved in their study that patients undergoing dialysis prior to TKA are significantly more likely to experience 30-day adverse outcomes [111].

Another important risk factor that interferes with the results and complications after knee replacement procedure is the lifestyle of patients. Sahota et al. evaluated the impact of smoking in a propensity score-match analysis, proving higher rates of deep surgical site infection and readmissions in the smokers cohort group [112]. Increased BMI is always an independent risk factor of complications and is associated with increased rates of reoperations in many cases, including patients who underwent TKA. With the increasing prevalence of obesity, the issue is gaining attention in the orthopedic community. Numerous research studies have shown that a higher BMI leads to increased aseptic loosening, implant revision or removal, acute kidney injury and cardiac arrest [113,114,115]. The risk level depends on the severity of obesity. Based on the findings of George et al., a potential BMI goal in weight management for obese patients could be established around 29–30 kg/m^2^ in order to decrease the risk of the most common TKA postoperative complications [116]. One of the possibilities in the management of BMI is bariatric surgery, which allows a patient to easily decrease their body mass. However, Martin et al. noticed in their research comparing two groups of patients who underwent TKA based on their bariatric operation status that the bariatric group had a higher risk of complications and worse survival free of re-operation compared with the pre-bariatric group [117]. On the other hand, Katakam et al. found that an underweight BMI was independently associated with an 8-times increased risk of mortality within 2 years following total joint replacement. Orthopedic surgeons should consider nutritional consultation and medical optimization in these high-risk patients prior to joint replacement surgery [118].

## 6. Conclusions

Considering the current state of knowledge and advancements in orthopedic development, the decision-making process regarding the selection of a specific prosthesis fixation method, whether it be cementless TKA or cemented TKA, necessitates careful consideration of various factors. Cemented TKA, long hailed as the traditional gold standard, has demonstrated commendable long-term success, providing a dependable approach for achieving joint stability and functional restoration. However, this equilibrium is disrupted when faced with specific patient demographics, particularly the younger, obese and more active populations, where the durability of cemented fixation may exhibit suboptimal performance. Advancements in surgical techniques and the development of cementless prostheses present a compelling alternative to traditional cemented TKA. These improvements acknowledge the evolving demographic landscape, suggesting that cementless TKA may be a viable and effective option. Our review indicates that presently there is no significant difference between cementless TKA and cemented TKA, emphasizing the importance of considering both methods in the context of individual patient needs and evolving orthopedic practices. In some cases, cementless TKA can be even better than cemented TKA. We should consider this type of fixation in younger patients because a cementless TKA provides better outcomes in this group. However, the cementless TKA also has disadvantages, like inferior range of motion, increased blood loss during surgery and higher pain scores after surgery than cemented TKA. Cemented TKA is a cheaper option, which is an important consideration for some groups of patients. As a result of our review, there is no universal method for fixing the knee prosthesis. The decision must be made individually for each patient with his or her participation and informed consent.

## Figures and Tables

**Table 1:** Comparison of types of fixation.

Study Type	Data Source	Population	Follow-Up	Conclusions	Period	Authors
Systematic review + meta-analysis of randomized controlled trials	Medline + Embase	6 studies—755 TKA (356 cemented, 399 cementless); mean age—62.5 years (range: 43–80); male-to-female ratio—1:3	8.4 years (2.0–16.6)	An overall significant difference in post-operative outcomes, including all-cause revision rate and knee function was not found. Increased postoperative pain in cementless group.	From the earliest to November 2018	Prasad et al. (2020) [68]
Systematic review + meta-analysis of randomized controlled trials	PubMed, Embase, Medline, Web of Science, and full Cochrane Library	6 studies—510 TKA (255 cemented, 255 cementless); age range 33–65	12 years (2–16.6)	Complication and survival rates were similar between groups. Cementless is superior to cemented TKA in young patients. Better clinical outcomes in cementless group.	From inception to July 2018	Wang et al. (2020) [76]
Prospectively followed and retrospectively analyzed trial	Surgeries performed by a single surgeon at single academic institution	261 patients (522 knees) who underwent bilateral simultaneous TKAs (one cemented, second cementless—order was randomized); mean age—62.5 years ± 5.5; 180 women: 81 men; BMI 27.2 ± 5.3	23.8 years (22–25)	Comparable outcomes and survivorship. Rate of survival at 25-year follow-up is 98% for cemented and 97% for cementless.	Between January 1995 and February 1998	Kim et al. (2021) [80]
An analysis from the National Joint Registry	National Joint Registry of England, Wales, Northern Ireland and Isle of Man	Propensity matched scoring techniques matched 44,954 cemented and cementless TKRs (22,477 cemented, 22,477 cementless)	7.2 years (SD 3.8)	Ten-year implant survival 95% in both groups (96% cemented vs. 95.5% cementless). The rates of revision for infection were lower in the cementless group, although rates of revision for pain were higher.	Between 1 January 2004 and 31 December 2018	Mohammad et al. (2021) [60]
Updated meta-analysis of comparative studies	PubMed, MEDLINE, Scopus and the Cochrane Central	18 studies—5222 patients (2734 cemented, 2343 cementless); mean age—64.4 years ± 9.4 (cemented) and 62.3 years ± 8.6 (cementless); female 48% (cemented) and 51.4% (cementless); BMI—33.2 ± 6 (cemented), 33.8 ± 6.6 (cementless)	107.9 months ± 30 (cemented) and 104.3 months ± 10 (cementless)	Comparable functional outcomes and reoperation rates. Cemented TKA showed less blood loss but a higher rate of manipulation under anesthesia and aseptic loosening.	Searched in January 2022 published over the past 15 years	Mercurio et al. (2022) [62]
Original research—retrospective review	PearlDiver patient record database. International Classification of Diseases and Current Procedural Terminology codes were used to identify patients who had undergone cemented and cementless TKAs and subsequent surgical revisions	324,508 patients, 312,988 (cemented, 11,520 cementless); females (57.67% cementless, 63.48% cemented); mean age—(64.02 years ± 9.2 cementless vs. 66.4 years ± 8.9 cemented)	90 days and 1 year after TKA	Decreased rate of I&D, one-component revision and arthroscopy with LOA in cemented group	From 2015 to 2019	Chiou et al. (2023) [59]
Retrospective review	Institutional review board. Current Procedural Terminology codes were used to identify patients who had undergone cemented or cementless TKA.	168 patients (80 cemented, 88 cementless); average age—57.94 (SD 14.13) in cemented group, 55.94 (SD 14.65) in cementless group; average BMI—33.98 (SD 5.43) in cemented group, 34.86 (6.90) in cementless group	More or equal to 2-year	Cemented group required fewer MUAs and had greater final ROM. Cementless group had lower tourniquet time.	Between January 2015 and June 2017	Edgar et al. (2023) [61]
Analysis of data from the National Joint Registry	National Joint Registry	Propensity matched scoring techniques matched 44,954 TKRs (22,477 cemented, 22,477 cementless)	3-month and 10-year fracture rates	Comparable rates of periprosthetic fracture at 3 months (0.02 cemented vs. 0.04 cementless) and 10 years (1.2% cemented vs. 1.4% cementless)	Between 1st January 2004 and 31st December 2018	Mohammad et al. (2023) [78]
Retrospective study	Procedures performed by 4 knee surgeons at 2 hospitals in the UK. During the period of this study, clinicians transitioned from use of the cemented UKR to the cementless UKR.	524 patients, 262 cemented and 262 cementless UKR	Patients were reviewed 5 years after UKR	UKR group had remarkably lower pain levels compared to TKR scores reported in the literature. Significantly less pain in cementless UKR group compared to cemented UKR group.	From 2006 to 2012	Rahman et al. (2023) [82]

## Data Availability

Publicly available datasets were analyzed in this study. This data can be found here: https://pubmed.ncbi.nlm.nih.gov/ (accessed on 25 February 2024).
